# Lack of definition of mathematical terms in ecology: The case of the sigmoid class of functions in macro‐ecology

**DOI:** 10.1002/ece3.7016

**Published:** 2020-11-23

**Authors:** Ugoline Godeau, Christophe Bouget, Jérémy Piffady, Tiffani Pozzi, Frédéric Gosselin

**Affiliations:** ^1^ INRAE UR EFNO Centre de Nogent‐sur‐Vernisson, Domaines des Barres Nogent‐sur‐Vernisson 45290 France; ^2^ INRAE UR MALY Centre de Lyon‐Villeurbanne Villeurbanne 69100 France

**Keywords:** biogeography, curve fitting, sigmoid curve shape, species–area relationship, species–resource relationship

## Abstract

Defining mathematical terms and objects is a constant issue in ecology; often definitions are absent, erroneous, or imprecise. Through a bibliographic prospection, we show that this problem appears in macro‐ecology (biogeography and community ecology) where the lack of definition for the sigmoid class of functions results in difficulties of interpretation and communication. In order to solve this problem and to help harmonize papers that use sigmoid functions in ecology, herein we propose a comprehensive definition of these mathematical objects. In addition, to facilitate their use, we classified the functions often used in the ecological literature, specifying the constraints on the parameters for the function to be defined and the curve shape to be sigmoidal. Finally, we interpreted the different properties of the functions induced by the definition through ecological hypotheses in order to support and explain the interest of such functions in ecology and more precisely in biogeography.

## Graphical abstract

The sigmoid class of functions is a mathematical entity commonly used in ecology but yet poorly defined. We propose a definition of this mathematical object as well as a list of functions commonly used in ecology belonging to this class. We finally discuss properties of sigmoid functions from the point of view of the underlying ecological hypotheses.

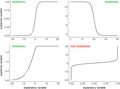

## 1 INTRODUCTION

Using well‐defined and uniform terms is a key point in science. Yet, one of the main criticisms that can be made in the science of ecology is the poor definition of terms and concepts or inconstant use within its community (Herrando‐Pérez et al., 2017; Kirk et al., 2018; Pickett et al., 2007). Many concepts do not yet have a consensual definition, and communication is therefore difficult. Furthermore, loosely defined concepts can cause not only an unstable expression of a scientific concept, but can also result in inconsistencies within the concept itself (e.g., Gosselin, 2001). This is why many articles have tried to highlight this problem and to establish precise definitions—that is “ecological niche” (Araújo & Guisan, 2006) or “ecological function” (Jax, 2005). However, the problem is not restricted to ecological concepts; it also concerns ecological domains (i.e., “ecological engineering,” cf. Gosselin, 2008) or certain terms and concepts used in ecology and borrowed from other sciences. This is the case for mathematical terms as, for example, the notions of extinction or demographic stochasticity (clarified in Gosselin, 1997; Lebreton et al., 2007). Reflections on mathematical definitions make it possible to conceptualize possibilities not yet foreseen (e.g., the importance of dependence between individuals within demographic stochasticity or uncertainty in Engen et al., 1998; McCarthy et al., 1994). In the present paper, we deal with the term “sigmoid” and propose a definition to overcome imprecision problems. Hereafter, we will call “sigmoid” the curve shape that can be represented by different functions, and the “sigmoid class of functions,” the class that contains these functions.

Ecologists often study relationships between two ecological variables (e.g., a biodiversity metric as a function of an environmental variable/predictor). Although the most often considered form of these relationships is linear, nonlinear forms have also been used (power, exponential, etc.), including sigmoidal forms. In ecology, sigmoidal relationships are generally implicitly used in logistic regressions. However, in the field of macro‐ecology and, in particular, in the study of species–area relationships (SARs), explicit sigmoidal forms occur fairly often. Indeed, a sigmoidal shape is very likely to emerge when species richness is related to the area in which the species were sampled (Preston, 1962). Many sigmoidal functions have been developed and used in a SAR context; however, they can also be applied to the study of relationships between biodiversity and a resource gradient other than available habitat area (species–resource relationships, or SReRs). Furthermore, the sigmoidal form of a relationship may prove useful for decision‐making in forest or conservation management. Indeed, certain characteristics of the curve can provide management targets like the inflection point or the upper asymptote (Ranius & Jonsson, 2007).

Over the years, numerous articles have been published which review the use of nonlinear functions, including sigmoids, in the field of biogeography and especially for SAR‐type relationships (Dengler, 2009; Tjørve, 2003, 2009; Williams et al., 2009). Unfortunately, no clear definition of the term sigmoid was provided in these publications.

Despite the frequent use of sigmoidal functions, in most cases, there is no proper, accessible definition of what exactly is meant by a “sigmoidal” shape. Classically defined as an S‐shape, the sigmoid may seem clear and that is the reason why it is so rarely defined. Yet, the precise characteristics of these curves are not formalized or made explicit. This absence of a clear definition results in a lack of harmonization between papers in ecology, and inconsistencies between articles, or even within one and the same article can ensue. For example, although most definitions include the presence of an upper asymptote (Veech, 2000), some authors like Mashayekhi et al. (2014), Triantis et al. (2012), Simaiakis et al. (2012) or Tjørve (2009) define functions (Extended power 1, Extended power 2, and Persistence 2) as sigmoidal though they do not have an upper asymptote; this contradicts the general idea of a sigmoid. The authors did not define the word sigmoid or explain what they meant under this designation in any way (with a definition, characteristics, or a reference) in their article. There is therefore a need to more explicitly define the sigmoidal class of shapes.

Our first goal was to assess the use of the term sigmoid in biogeography studies and highlight the lack of a clear definition. Then, we propose a definition of the term so that its use in the literature is harmonized and no longer confusing. Finally, we justify the definition in relation with ecological theory and we highlight the implications and advantages of this new definition. The two underlying questions are as follows: What characteristics should sigmoid curves exhibit? What functions can be included in the sigmoid class?

## 2 AN OBVIOUS LACK OF A CLEAR DEFINITION

The word “sigmoid,” composed of “sigma” and “eidos” (*sigmoeidḗs* in ancient Greek), means something that has the form of the capital letter sigma (Σ). The term sigmoid is more generally defined as an S‐shaped curve. Yet, these descriptions, in addition to being vague, are not accurate since the form of an S (or a Σ) is impossible in mathematical curves described by functions. In fact, if we apply an S form to mathematical curves, we notice that we obtain two or three values of *f*(*x*) for one *x*, which is impossible according to the very definition of a function (in its classical, usual definition in mathematical analysis). Moreover, the representation of an S‐shaped curve excludes forms that should logically be part of sigmoid curves such as decreasing sigmoid curves.

Given this intrinsic difficulty with the notion of sigmoid, we investigated how authors in ecology have used and defined this term. Sigmoid curves are explicitly used to describe various phenomena studied in ecology like dose response, exposure response, stimulus response, density dependence, and species accumulation. We chose to focus on a part of these phenomena by restricting ourselves to the field of biogeography with species–area relationships (conventionally abbreviated as SARs) and species response to ecological gradients within species–resource relationships (abbreviated here as SReRs).

In July 2020, we conducted a literature survey via the Web of Science, searching for articles released before 2020 in the category “Ecology,” with the following keywords: (“biogeography” OR “SAR” OR “species‐area” OR “species area” OR “species‐resource” OR “species resource” OR “species response” OR “species‐response”) AND (“nonlinear” OR “non‐linear” OR “non linear” OR “sigmoid*” OR “logistic” OR “S‐shape*” OR (“asymptot*” AND "inflection point") OR “density depend*” OR “density‐depend*” OR “accumulation curve” OR “species accumulat*”).

We extracted a list of the articles resulting from this survey and calculated the proportion of articles in which authors used a sigmoid function or were interested in a sigmoidal form of relationship in a statistical model. In order to determine whether the article explicitly uses or discusses a sigmoid function, without having to read it in its entirety, we proceeded in three stages: (a) reading the abstract entirely or partially (in order to have an idea of the content of the article); (b) flying over the article in search of tables with functions, figures, or equations which would be sigmoidal and, if necessary, reading the associated paragraph and/or legend; (c) for articles where the PDF allowed it, searching for the keywords: "sigm," "logist," "non‐linear," and "s‐shape" (both written in different ways), as well as the other keywords that seemed relevant when reading the abstract (e.g., density dependence or species accumulation). If some articles using a sigmoid function have been able to pass through the mesh of the net with this method (in particular using a function other than logistic), it will be articles not using a “sigmoid” word to characterize the relation described, thus, potentially biasing the results in favor of a larger proportion of articles using the word sigmoid. We completed this list with 13 articles of our personal knowledge from the field of biogeography and using a sigmoid function, which did not emerge using the survey on the Web of Science. In order to represent how the authors define their sigmoid function, we recorded which words were used from the list of keywords provided above. Then, in order to identify the use and understanding of the specific term sigmoid by article authors, for the selected articles using a sigmoid function, we classified them into four different categories as follows:

1. CATA: The authors do not use a term to define the function or the shape of the curve.
2. CATB: The authors only use an imprecise term to define the function or the shape of the curve (e.g., S‐shape).
3. CATC: The authors use the name of the function (e.g., logistic), without referring to the sigmoid class/form.
4. CATD: The authors use a word from the “sigmoid” word family.

Finally, for articles using a word from the “sigmoid” word family (CATD), to report the proportion of articles incorporating a definition of this word, we have classified the articles in the following four subcategories:

1. subD1: The authors do not define “sigmoid”
2. subD2: The authors only cite a reference to define “sigmoid”
3. subD3: The authors partially define “sigmoid”
4. subD4: The authors clearly define “sigmoid”

**FIGURE 1:**
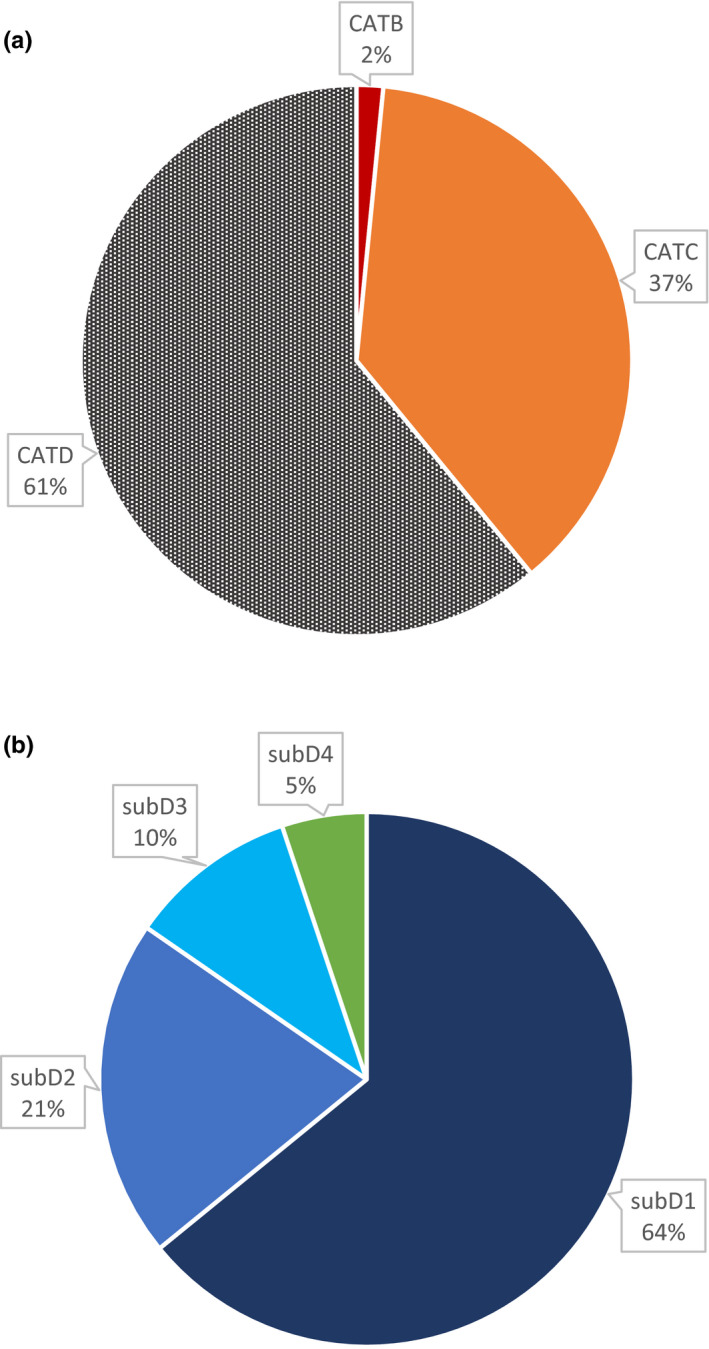
Distribution of the articles resulting from the survey of Web of Science (excluding articles on binary data), in the different described categories (a) and subcategories of the category D (b). The categories are defined as follows: CATB = the authors only use an imprecise term to define the function or the shape of the curve (e.g., S‐shape); CATC = the authors use a precise term, for example, to name the function (e.g., logistics), without referring to the sigmoid class/form; CATD = the authors use a word of the same family word as “sigmoid”; subD1 = the authors do not define sigmoid; subD2 = the authors only cite a reference to define sigmoid; subD3 = the authors partially define sigmoid; subD4 = the authors clearly define sigmoid

The articles using logistic regression on binary data were more numerous than on nonbinary data (99 vs. 64) and very rarely acknowledge that the underlying function is of sigmoidal form (Figure S1). The classification in categories for all the articles resulting from our bibliographic research (plus additions) seemed therefore to be strongly influenced by the 99 papers on binary data (Figure S2, Table S2). In the rest of the article, we analyzed in more detail the behavior of articles using sigmoid functions on nonbinary data (64 articles, Figure 1, Table S1). We also performed a GLM to explore whether the distribution in the different subcategories of these 64 articles depended on the publication date, in other words, if we could observe a change in the authors' desire to define the word sigmoid over the years. The majority of the articles (61%) were using the word sigmoid (or any word of the same family) to describe the function used (CATD). Thirty‐seven percent of the articles were only referring to the name of a sigmoid function (CATC) and 2% used a very imprecise word to designate the function (CATB). There was no apparent change in the incorporation of a definition of sigmoid in the articles using the word sigmoid over the years (GLM: *p*‐value = 0.593). Over the 39 articles using the word sigmoid, only a few authors were taking the time to properly define what the word sigmoid was implying (subD4 = 5%). The vast majority did not define what they meant by sigmoid (subD1 = 64%). What was quite surprising was that some authors created new sigmoid functions and stated that their functions have a sigmoidal form, but they never evoke the characteristics implied by this form and included in their function (e.g., Kobayashi, 1976). In other cases, some authors were partially defining the notion (or formulating some characteristics associated with sigmoid function or shape—subD3 = 10%) or pointing to references (subD2 = 12%) in order to help readers understand what they meant by sigmoid. However, these definitions were incomplete, or fragmented, as well as the definitions contained in the cited references. Unclear definition, or imprecisely characterized functions, can lead to confusion or conflicting conceptions for the reader.

For instance, Preston (1962) proposed a descriptive definition of the shape of the sigmoid curve, which gives us an idea of the form but without specifying its properties: “it began at a low slope, steepened considerably, and then became less steep.” Tjørve (2003, 2009), on the other hand, does not give a definition of the sigmoid curve, but does mention some of its characteristics when describing the functions he considers in his study. In Tjørve's papers (), the characteristics common to all sigmoid functions include: (a) the presence of an upper asymptote, (b) a lower j‐shape (probably implying a lower asymptote), and (c) the presence of an inflection point. Tjørve (2003, 2009) also mentions two characteristics which vary among different sigmoid functions: symmetry around the inflection point, which may or may not exist; and the positions of the inflection point and of the asymptote.

Furthermore, in addition to being incomplete, these "definitions" may present other problems that impede understanding. This is the case when mathematical terms characterizing a mathematical object, here the sigmoid curve, are incorrectly used. For example, some authors erroneously define their sigmoid functions as “convex” (Gentile & Argano, 2005; Tjørve, 2003,2009). Indeed, in mathematics, a curve (or function) is “convex” (or having an overproportional increase) if, for any two points *A* and *B* of the curve, the segment [*AB*] is entirely situated above the curve. Conversely, a concave function is the opposite of a convex function (*f* is concave if and only if −*f* is convex). A concave (or having an underproportional increase) curve (or function) is therefore a curve for which, for any two points *A* and *B* of the curve, the segment [*AB*] lies entirely below the curve. Yet, some studies make no distinction between the two curves and use “convex” for both convex and concave forms (Tjørve, 2012), then distinguish them with the mentions “downward” or “upward.” Usually, given the properties attributed to the curves defined as convex, the term concave, rather than convex, is clearly the correct term. For example, what Tjørve (2009) described as a "constantly decelerating" convex curve is actually concave, and what he defined as a "J‐shape" would correspond to the convex part of the sigmoid curve. This error is common since convex and concave shapes are often respectively described as a hump and a hollow (from the definition of a convex set), which can lead to confusion. Therefore, though the study is very interesting, the discourse is blurred by terms that are confusing (as also pointed out by Dengler, 2009). Consequently, we suggest using mathematical definitions and terms, so that all researchers will refer to the same definition of sigmoid curves.

If one moves away from the literature in ecology, we find that few definitions are easily accessible even in statistical literature. Hill and Lewicki (2006) propose one such definition in their glossary: A sigmoid function is “an S‐shape curve, with a near‐linear central response and saturating limits” (p. 724). This definition, which includes the notion of an S‐shape discussed above, makes it possible to understand the general shape and to accept different forms, but they are not necessarily very clear on which forms are included or excluded when we speak of a sigmoid, and the properties of the functions are not precise. Menon et al. (1996) also start by defining the sigmoid curves as S‐shaped; then, the authors define two subclasses of sigmoids: (a) simple sigmoids are “odd, asymptotically bounded, completely monotone functions in one variable,” and (b) hyperbolic sigmoids are “a proper subset of simple sigmoids and a natural generalization of the hyperbolic tangent.” Although detailed, notably when characterizing certain functions, the two defined classes do not integrate all the possible sigmoidal forms; for example, “odd” excludes asymmetric curves and curves that do not intersect the origin.

To sum up, very few definitions of sigmoid functions are available in the ecological literature, and they are usually vague, or based on only certain characteristics, or can even contain errors. Therefore, it seems clear that the lack of a time‐honored definition, or the use of unstable definitions, can lead to difficulties in producing studies and articles. This is particularly true for bibliographic research and for young researchers and students (PhD or Masters students) who are still forging their knowledge (Herrando‐Pérez et al., 2017). It can also sometimes distort communication among collaborators. For example, within our own research group, differences of wording regarding the properties of different curves have surfaced, with misunderstandings of what is meant by “convex” and “concave.”

The shape of the curve must be well integrated during its use in order to properly interpret the results. As put forward by Fattorini et al. (2012), Medellín and Soberón (1999) used a sigmoid model on their data, and then, in order to ensure fit with a logarithmic model, they chose to exclude some of the data corresponding to the first part of the sigmoid curve (where the slope is smaller). In this specific case, removing the beginning of the gradient results in a poor estimation of the variation of the slope along the gradient. In that respect, Fattorini et al. (2012) point out that Medellín and Soberón (1999) should not have manipulated the data and should have retained a model that fits the entire dataset, the data represented by the first part of the curve being just as important from an ecological point of view as the data represented by the rest of the curve. In fact, the first part of the curve could reflect various ecological mechanisms that deserve to be studied such as—to name but two: sampling problems or biological functions in action (i.e., another limiting factor, exclusions). Through this example, it becomes obvious that if the sigmoid curve shape and its implications are not acknowledged or defined well enough in the mind of the ecologist, he may end up missing important patterns or making wrong assumptions.

## 3 PROPOSAL OF A CLEAR DEFINITION

Although the definition on Wikipedia is globally correct (Wikipedia, no date), this website cannot be used as a reference since the page can be modified at any time, making the definition unstable. We have therefore decided to propose a definition, which is stable, understandable for ecologists, and as complete as possible (including as many cases as possible) in this paper. For this purpose, we first looked at the characteristics of the functions used in the literature.

Ultimately, a sigmoid curve is a curve described by a real‐valued, univariate function (a function f of a unique real‐valued variable *x* that takes real values *y* = *f*(*x*)), defined over the whole set of real numbers, and which is continuous, infinitely differentiable, monotonic (always either increases or decreases), has at least one inflection point and is bounded on the *Y*‐axis. The term “inflection point” refers to the point where the curve shifts in convexity: from convex to concave or vice versa. The change in slope is continuous and should therefore be distinguished from the term “breakpoint” used by ecologists, which, although we did not find a precise mathematical definition, seems to refer to a noncontinuous function (e.g., in change point models, Muggeo, 2003; Quandt, 1958).

Consequently to the description given above, a sigmoid curve has the following inherent characteristics: (a) has an upper and a lower asymptote if (*x*) varies over the set of real numbers; (b) can increase (starting with the lower asymptote and finishing with the upper asymptote, with a positive slope between them, Figure 1, 2.a) or decrease (starting with the upper asymptote and finishing with the lower asymptote, with a negative slope between them, Figure 1, 2.b); and (c) can be symmetrical or not around the inflection point or points (Figure 1, 2.c).

We extend the definition given above to two other cases where the explanatory variable (*x*) is defined on the set of real positive numbers (*x* ≥ 0) and (a) *f*(*x*) is a function of (*x*) over the entire set of real numbers and has a sigmoid curve, or (b) the above definition for the sigmoid curve applies to *f*(*x*) as a function of (*x* ≥ 0) except for the requirement that *f*(*x*) is defined over the entire set of real numbers. Indeed, in island biogeography, the function never occurs with negative *x*‐values (since area cannot be negative). In this case, the sigmoid curve has only one of the two asymptotes. Further note that the sampled gradient may not include the inflection point or result in a function that comes close to the asymptote(s) and therefore may not give a full sigmoidal curve shape on the sampled gradient, the function nevertheless belonging to the class of sigmoid functions (e.g., Godeau et al., 2020; Tjørve, 2009). Even after extension, however, our definition does not include the case where (*x*) is bounded on both sides and therefore possesses neither of the two asymptotes (He & Legendre, 2002). Note that *f*(*x*) as a function of (*x*) can have a sigmoidal form without *f*(*x*) as a function of log(*x*) or *f*(exp(*x*)) as a function of (*x*) being sigmoidal too, and vice versa.

**FIGURE 2:**
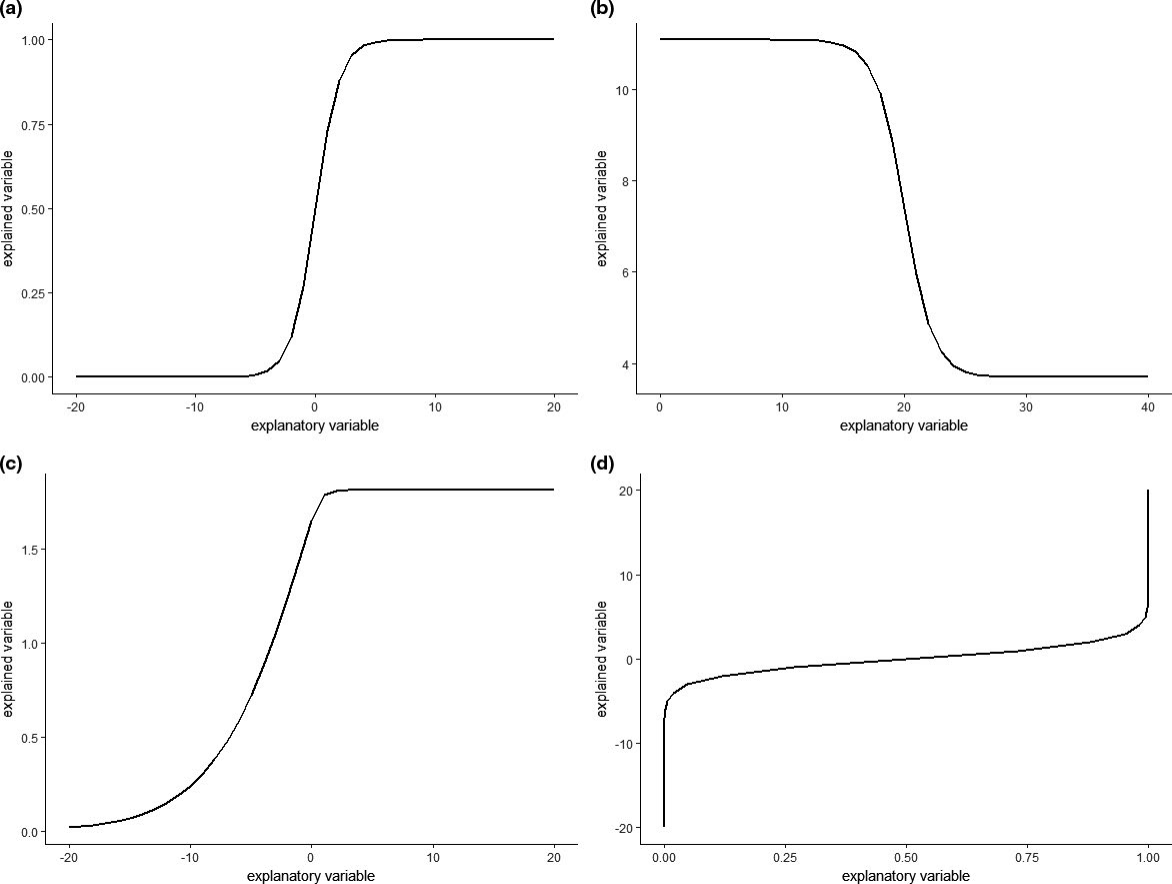
Some possible forms of sigmoids and inverse sigmoids. (a) Simple logistic function, (b) decreasing sigmoid, (c) asymmetric increasing sigmoid, and (d) increasing inverse sigmoid

The class of sigmoid functions includes the functions which, for the given parameters, meet the above definition. The same function may or may not belong to the sigmoid class depending on the value of its parameters (as also notified by Gao & Perry, 2016; Triantis et al., 2012). To return to a previous example, the Chapman‐Richards function belongs to the sigmoid class if *c* > 1. For other values of *c*, the function does not belong to the sigmoid class. This characteristic leads to a flexibility of the functions (giving curves which can be sigmoid, convex, concave, or linear) which is under‐appreciated.

The sigmoid class can be divided into two subclasses: (a) simple sigmoids, containing the functions that give curve shapes with a single inflection point, and (b) multiple sigmoids containing functions that give curve shapes with several inflection points (i.e., a double sigmoid could fit the phenomenon described in Figure 6 in Lomolino, 2000). There must always be an odd number of inflection points in order to keep the two asymptotes on the *Y*‐axis.

Based on the definition of the sigmoid class that we propose above, we inventoried the classical SAR or SReR functions selected from the prospect we conducted that belong to the simple sigmoid class, at least for some parameter values (see Table 1). We also described their characteristics, placing special emphasis on the constraints imposed on the parameter values or explanatory variable to ensure that the function is mathematically defined, is suitable in macro‐ecology, and does indeed have a sigmoidal form. We also provide the coordinates of the inflection point, so that readers can distinguish between functions that are sigmoidal only when the whole set of real values for the explanatory variable is considered (i.e., functions with a negative abscissa value of the inflection point) and those that are sigmoidal even when the abscissa values are positive. Having a well‐established definition of the sigmoid curve and understanding the constraints imposed on the parameter values of the functions, which produce sigmoid curves allow us to better apprehend under which conditions a sigmoid function is adapted when one wishes to apply it to a dataset. For example, the Chapman‐Richards function is defined only for (*x* ≥ 0) and the curve obtained will only be of sigmoid shape when (*c* > 1) (see Table 1). Another, more extreme, example combines these two limitations: the persistence 2 function. In fact, this function is sigmoid only if (*x* > 0), (*b* = 0), and (*c* > 0).

**TABLE 1:** Some characteristics of sigmoidal functions used or usable in a biogeography context

	Formula	Constraints on parameters to be defined and relevant to macro‐ecology	Further constraints required to be in the sigmoid class	Inflection point	Symmetry around the inflection point	Lower asymptote	Intersects origin	Direction of the relationship	Reference
Common logistic	*f*(*x*) = *a*/(1 + exp(−*b* * *x* + *c*))	*a* > 0	/	*x* = *c*/*b* *y* = *a*/2 In other terms: *y* = 50% of the upper asymptote	Point symmetry	Zero	No	Increasing (if *b* > 0) or decreasing (if *b* < 0)	Ratkowsky (1990)
Cumulative Gaussian	*f*(*x*) = *a* * Φ(*b* * (*x* − *c*)), where Φ is the cumulative function of the standard Gaussian probability distribution	*a* > 0	/	*x* = *c* *y* = *a*/2 In other terms: *y* = 50% of the upper asymptot	Point symmetry	Zero	No	Increasing (if *b* > 0) or decreasing (if *b* < 0)	McCullagh and Nelder (1989)
Arctan	*f*(*x*) = *a* * (arctan(*b* * (*x* − *c*) + *π*/2)/*π*	*a* > 0	/	*x* = *c* *y* = *a*/2 In other terms: *y* = 50% of the upper asymptot	Point symmetry	Zero	No	Increasing (if *b* > 0) or decreasing (if *b* < 0)	Fox and Vasseur (2008)
U‐Bertalanffy	*f*(*x*) = *a* * (1−1/3 * exp(−9 * *b* * (*x* − *c*)/4))^3^	*a* > 0	/	*x* = *c* *y* = *a* * (2/3)^3^ In other terms: *y* = 29.6% of the upper asymptote	Asymmetric	Zero	No	Increasing (if *b* > 0) or decreasing (if *b* < 0)	Vrána et al. (2019)
Gompertz	*f*(*x*) = *a* * exp(−exp(−*b* * *x* + *c*))	*a* > 0	/	*x* = *c*/*b* *y* = exp(−1) * *a* In other terms: *y* = 36.8% of the upper asymptote	Asymmetric	Zero	No	Increasing (if *b* > 0) or decreasing (if *b* < 0)	Ratkowsky (1990)
U4 model	*f*(*x*) = *a*/(1 + 3 * exp(−4^(4/3)^ * *b* * (*x* − *c*)))^(1/3)^	*a* > 0	/	*x* = *c* *y* = *a*/4^(1/3)^ In other terms: *y* = 63.0% of the upper asymptote	Asymmetric	Zero	No	Increasing (if *b* > 0) or decreasing (if *b* < 0)	Vrána et al. (2019)
Extreme value	*f*(*x*) = *a* * (1 − exp(−exp(*b* * *x* + *c*)))	*a* > 0	/	*x* = −*c*/*b* *y* = [1‐exp(−1)] * *a* In other terms: *y* = 63.2% of the upper asymptote	Asymmetric	Zero	No	Increasing (if *b* > 0) or decreasing (if *b* < 0)	Williams (1995, 1996)
Chapman‐Richards	*f*(*x*) = *a* * (1 − exp(−*b* * *x*))*^c^*	*a* > 0, *x* ≥ 0, *c* > 0, *b* > 0	*c* > 1	*x* = log(*c*)/*b* *y* = *a* * (1 − 1/*c*)*^c^*)	Asymmetric	/(irrelevant since *x* is non‐negative)	Yes	Only increasing	Ratkowsky (1990)
Cumulative Weibull distribution	*f*(*x*) = *a* * (1 − exp(−*b* * (*x^c^*)))	*a* > 0, *b* > 0, *x* ≥ 0	*c* < 0 or *c* > 1	*x* = ((*c* − 1)/(*b* * *c*))^(1/^ *^c^* ^)^ *y* = *a* * (1 − exp(−1 + 1/*c*))	Asymmetric	/(irrelevant since *x* is non‐negative)	Yes (if *c* > 0)	Increasing (if *c* > 0) or decreasing (if *c* < 0)	Weibull (1951)
Morgan‐Mercer‐Flodin (MMF)	*f*(*x*) = *a* * (*x^c^*)/(*b* + (*x^c^*))	*a* > 0, *b* > 0, *x* ≥ 0 (with *f*(0) = *a* if *c* < 0 to be continuous)	*c* > 1 or *c* < (−1)	*x* = ((*c* − 1) * *b*/(*c* + 1))^(1/^ *^c^* ^)^ *y* = *a* * (1/2 − 1/(2 * *c*))	Asymmetric	/(irrelevant since *x* is non‐negative)	Yes	Increasing (if *c* > 0) or decreasing (if *c* < 0)	Morgan et al. (1975)
Cumulative beta‐P distribution	*f*(*x*) = *a* * (1 − (1 + (*x*/*c*)*^d^*)^(−^ *^b^* ^)^)	*a* > 0, *x* ≥ 0, *c* > 0, *b* > 0	*d* > 1 or *d* < (−1/b)	*x* = *c* * ((−d + 1)/(−*b* * *d* − 1))^(1/^ *^d^* ^)^ *y* = *a* * (1 − (1 + (−*d* + 1)/(−*b* * *d* − 1))^(−^ *^b^* ^)^)	Asymmetric	/(irrelevant since *x* is non‐negative)	Yes (if increasing, *d* > 0)	Increasing (if *d* > 0) or decreasing (if *d* < 0)	Mielke and Johnson (1974)
U_Richards	*f*(*x*) = *a* * (1 + (*d* − 1) * exp(−*b* * (*x* − *c*)/*d* ^(^ *^d^* ^/(1−^ *^d^* ^)^))^(1/(1−^ *^d^* ^))^	*a* > 0, *d* > 0 and *d* different from 1	/	*x* = *c* *y* = *a* * (1 + (*d* − 1))^(1/(1−^ *^d^* ^))^	Asymmetric (except if *d* = 2)	Zero	No	Increasing (if *b* > 0) or decreasing (if *b* < 0)	Vrána et al. (2019)

Note

that models II and III in Huisman et al. (1993), denoted as *f*(*x*) = *M* * (1/(1 + exp(*a* + *b* * *x*))) and *f*(*x*) = *M* * (1/(1 + exp(*a* + *b* * *x*))) * (1/(1 + exp(*c*))), are particular cases of the Common Logistic Function with, respectively, parameter (*a*) not estimated, and with parameter (*a*) estimated but with a given maximum value. The Archibald Logistic Function (Archibald, 1949), denoted as *f*(*x*) = *a*/(*b* + *c^x^*), is equivalent to the Common Logistic Function with (*b*), (*c*), and (*a*) in the Common Logistic Function, respectively, equal to (−log(*c*)), (−log(*b*)), (*a*/*b*) in the Archibald Logistic Function. The He‐Legendre Function (Lomolino, 2000, respectively), denoted as *f*(*x*) = *a*/(*b* + (*x* ^(−^ *^c^* ^)^)) (resp. *f*(*x*) = *a*/(1 + (*b* ^log (^ *^c^* ^/^ *^x^* ^)^))), is equivalent to the Morgan‐Mercer‐Flodin Function with (*a*) and (*b*) of the MMF, respectively, equal to (*a*/*b*) and(1/*b*) in the He‐Legendre Function ((*b*) and (*c*) of the MMF, respectively, equal to *b* ^log (^ *^c^* ^) and log(^ *^b^* ^)^ in the function used by Lomolino). The type III Holling function (Holling, 1959a, 1959b), denoted as *f*(*x*) = *ax* ^2^/(*b* ^2^ + *x* ^2^), is equivalent to the MMF, with (*c*) and (*b*) in the MMF, respectively, equal to (2) and (*b* ^2^) in the Holling III Function.

Threshold functions—functions with a constant value below a threshold and another constant value above it (Toms & Villard, 2015)—are a class of functions that can be close to sigmoid functions (especially to limits of sigmoid functions when the maximum slope of the function tends to infinity) but that are no included in the sigmoid class because they are not continuous and not infinitely differentiable, and thus make it difficult to define an inflection point as classically done with the second derivative. Another class of functions that is close to the sigmoid class is the class of inverse sigmoid functions. These are bounded on the *X*‐axis and do not have an asymptote over the *Y*‐axis (Figure 1, 2.d). These functions have no biological reality in SReR and SAR and are not members of the sigmoid class as we define it. Other curves defined as sigmoid by some authors do not meet the requirements of our definition either, for example, “sigmoid curves […] free of upper asymptotes” (Tjørve, 2012).

## 4 ECOLOGICAL JUSTIFICATIONS AND IMPLICATIONS OF SIGMOID CURVE CHARACTERISTICS

Although some characteristics of the sigmoid definition are justified mainly by mathematical considerations, many can be related to ecological hypotheses or considerations. First, the presence of an inflection point represents the tipping point between the beginning and the end of the gradient. At the beginning of the gradient the more *X* increases, the more the advantage conferred by *X* is important. At the end of the gradient the advantage conferred by *X* allows less and less to overcome other limitations. In SARs, the use of sigmoid curves with this inflection point is justified by the following statement by Lomolino (2000): “with richness remaining relatively low and apparently independent of area for the smaller islands, increasing rapidly to rise through an inflection point for islands of intermediate size, and then asymptotically approaching, or leveling off at the richness of the species pool for the largest islands.” Many other fields of ecology are interested in models that can depict such a pattern (e.g., ecophysiology: Paine et al., 2012). Continuity and differentiability would allow us to formulate hypotheses not only on the mean value of the response variable, but also on the speed (first derivative) or acceleration (second derivative) of the relationship between the response variable and the gradient being studied, which however has not been done so far. The pattern depicted by Lomolino for SARs might have led us to define sigmoid curves only as increasing curves. Yet, we expect that in some areas of ecology the reversed situation might occur and that such patterns would indeed fall into the domain of the sigmoid curve. For example, still in biogeography, a decreasing sigmoid was considered in species–isolation relationships (Hachich et al., 2015). More generally in ecology, the decreasing sigmoidal curve can be used in the case where the gradient studied has a negative effect on the response variable (e.g., Morante‐Filho et al., 2015).

Second, the existence of asymptotes is also very much related to considerations from ecology. The upper asymptote, implying a threshold above which the mean of the response variable (*y*) cannot go, theoretically reflects the Liebig law of the minimum in ecophysiology and ecology (Austin, 2007; Paris, 1992). In this case, the studied predictor would be the first limiting factor, and an increase in this limiting factor would lead to an increase in the explained variable. Then, upon reaching the asymptote, the predictor would no longer be limiting; instead, another unmeasured environmental factor would take over, though its influence would be insufficient to make the explained variable increase any further. More particularly in the study of SAR, as species richness increases with area and decreases with geographical isolation, an upper asymptote can emerge at very large areas when the number of species equals the number of potential species in the regional species pool. This number can be obtained into smaller areas, when immigration increases (Kadmon & Allouche, 2007; MacArthur & Wilson, 1967).

Inversely, the presence of a lower asymptote implies that the mean of the response variable cannot be lower than this asymptote. The existence and value of such an asymptote can often be related to the conjunction of the monotonic relationship, the nature of the variable considered, and the nature of the system under study. In studies focusing on the response of a single species, the lower asymptote is therefore usually zero (e.g., Huisman et al., 1993). However, when studying community response, often a lack of resources does not necessarily imply a total loss of species richness (e.g., when studying a system where species are mobile). In such cases, a logistic function where *f*(*x*) is a function of log(*x*), whose lower asymptote is necessarily located at zero (*y* = 0) and is not actually adapted (Godeau et al., 2020).

The third component of our definition is asymmetry of the curve. Symmetric sigmoid curves, like the common logistic function, are widely used, but more for their ease of modeling than for their underlying ecological theory. In Generalized Linear Models (GLMs), both common link functions (logit and probit) imply symmetrical sigmoid shape through their inverse. However, for bell‐shaped curves, Austin (1976) stated: “there is no a priori reason to assume that organisms' responses should follow such a symmetrical curve,” and it is very likely that this remark is also true for the sigmoid curves. Diverse phenomena can explain asymmetrical curves (Austin, 1990; Austin & Gaywood, 1994 for phyto‐ecology) and theoretically supported asymmetry can also appear with sigmoidal curves (e.g., Lim et al., 1998). Thus, there is a third canonical link function for GLMs (the complementary log‐log function) which allows asymmetry through its inverse, and which can be derived from assumptions regarding, for example, survival rates, which is asymmetric.

More generally, the overall shape of the sigmoid curve is well justified in a wide variety of cases (as for example Type II model to identify habitat thresholds in Yin et al., 2017; or sigmoidal curves for biodiversity–ecosystem functioning relationships in Maureaud et al., 2020). However, a sigmoid curve can take several forms (in particular concerning the position of the inflection point), depending on the function used and the value of its parameters, it is important to also dwell on this aspect when adjusting to the data (e.g., Vrána et al., 2019).

## 5 CONCLUSION AND PERSPECTIVES

Our literature prospection points out the lack of a clear, stable, universally accepted definition of the sigmoid class of functions in ecology. Some aspects of sigmoid curves are typically ignored (symmetry, direction of the relation, etc.). We also found cases of misuse of convexity to define a curve or a function.

As Jeremy Fox stated “words are imprecise, and so purely verbal models and verbal arguments often are ambiguous or even invalid, even if apparently supported by empirical data (like Elton's verbal arguments about why diversity and complexity beget stability). Mathematics has the virtue of forcing precise definitions of terms, precise and complete specification of assumptions, and rigorous derivation of conclusions” (Fox, 2011). It is therefore unfortunate to accept vague verbal definitions (such as “S‐shape” or “J‐shape”) when one is using a term derived from mathematics.

That is why we have proposed a definition that we hope will allow for better harmonization of what is meant by the term “sigmoid” when describing a curve or a function. In addition to clearly formulating the concept, our definition allows various functions to be united under the same banner (sigmoid class, presented in Table 1). This definition also excludes some functions that were previously considered to belong to the sigmoid family and which, in our opinion, should not be defined as such (sigmoid without an upper asymptote or inverse sigmoid).

Having clear definitions makes it possible to more clearly reflect on the underlying concepts and theories implied by the functions available, and to visualize the most appropriate form of curve to adopt according to the ecological context. After defining and reflecting on the lower asymptote and asymmetry, the researcher naturally questions the choice of link function in the context of logistic regressions. Classically, users of such tools choose canonical link functions such as the logit or the probit function. The inverse of these two functions, on which the regression relies, belong to the sigmoid class but they are symmetric around the inflection point and they have prespecified minimum and maximum asymptotes (0.0 and 1.0, respectively). However, the inherent properties of such link functions could have strong ecological limitations, which would restrict their use in some cases. For example, having a maximum of 1.0 (meaning almost sure presence) along the gradient does not reflect biological situations where, even if local habitat conditions are optimal for the organism, the organism could be absent (e.g., due to dispersal limitation inside a metapopulation; Hanski & Gilpin, 1997). Thus, a more flexible use of the sigmoid function in these logistic regressions can prove to be of great use (Godeau and Gosselin, Eide et al., 2012; In prep.). Along the same lines, sigmoid and logistic functions are sometimes confused with each other, whereas the latter is nothing more than a particular type of sigmoid (e.g., Hunsicker et al., 2015). Such confusion may prevent researchers from considering other families of functions that fall into the sigmoid class without being logistic.

In other papers, we aim to develop a sigmoid function that incorporates the characteristics retained in this paper: first in an SReR context (Godeau et al., 2020) and second in binomial logistic regressions. Such development of the sigmoid class might be of more general use in ecology, for example, by broadening the scope of possibilities in binomial logistic regressions.

Finally, we hope that in future papers, authors who define a new sigmoid function, or use an already existing one, will take the time to specify the properties of the function and to clearly mention their implications and/or justifications in ecological terms.

## CONFLICT OF INTEREST

The authors have no conflict of interest to declare.

## AUTHOR CONTRIBUTIONS

**Ugoline Godeau:** Conceptualization (equal); data curation (lead); formal analysis (lead); investigation (equal); methodology (equal); writing – original draft (lead). **Christophe Bouget:** Conceptualization (supporting); writing – review and editing (equal). **Jérémy Piffady:** Conceptualization (supporting); writing – review and editing (equal). **Tiffani Pozzi:** Conceptualization (supporting); writing – review and editing (equal). **Frédéric Gosselin:** Conceptualization (equal); funding acquisition (supporting); investigation (equal); methodology (equal); supervision (lead); writing – original draft (supporting).

## Supporting information

Table S1

Table S2

Appendix S1

## Data Availability

No data were used for this manuscript.
