# Supplementary material for: Lack of definition of mathematical terms in ecology: The case of the sigmoid class of functions in macro‐ecology
Source: Ecol Evol. 2020 Nov 23;10(24):14209–20. doi: 10.1002/ece3.7016 (PMC7771130; doi:10.1002/ece3.7016)
Supplement: Supplementary file 3 — Appendix S1 [file ECE3-10-14209-s003.docx]

# APPENDIX S1

Figure S1 : Distribution of the articles on binary data, resulting from the survey of Web of Science, in the different described categories (A) and sub-categories of the category D (B). The categories are defined as follows: CATC = the authors use a precise term, for example to name the function (e.g. logistics), without referring to the sigmoid class / form; CATD = the authors use a word of the same family word as “sigmoid”; subD1= the authors do not define sigmoid; subD3 = the authors partially define sigmoid.

Figure S2: Distribution of all the articles, resulting from the survey of Web of Science, in the different described categories (A) and sub-categories of the category D (B). The categories are defined as follows: CATB = the authors only use an imprecise term to define the function or the shape of the curve (e.g. S-shape); CATC = the authors use a precise term, for example to name the function (e.g. logistics), without referring to the sigmoid class / form; CATD = the authors use a word of the same family word as “sigmoid”; subD1= the authors do not define sigmoid; subD2 = the authors only cite a reference to define sigmoid; subD3 = the authors partially define sigmoid; subD4 = the authors clearly define sigmoid.
